# Assessment of prognosis by physicians involved in work disability evaluation: A qualitative study

**DOI:** 10.1371/journal.pone.0212276

**Published:** 2019-02-08

**Authors:** René J. Kox, Jan L. Hoving, Jos H. Verbeek, Maria J. E. Schouten, Carel T. J. Hulshof, Haije Wind, Monique H. W. Frings-Dresen

**Affiliations:** 1 Amsterdam UMC, Location AMC, Department Coronel Institute of Occupational Health, Amsterdam Public Health Research Institute, Amsterdam, The Netherlands; 2 Research Center for Insurance Medicine (KCVG), Amsterdam, The Netherlands; 3 Finnish Institute of Occupational Health, Kuopio, Finland; Medical University Graz, AUSTRIA

## Abstract

**Background:**

Assessment of prognosis of work functioning is a challenging aspect of work disability evaluations. To gain insight into this process, we conducted a qualitative study to determine the aspects considered and the difficulties, needs and potential solutions affecting the prognosis assessment by physicians performing disability evaluations.

**Methods:**

In-depth, semi-structured individual interviews were conducted with 20 physicians performing disability evaluations for the Dutch social security institute: the national institute for employee benefit schemes. Verbatim transcripts were independently analyzed by two researchers using MAXQDA software until significant themes emerged and data saturation was achieved.

**Results:**

The responses that emerged from the interviews were clustered in three primary themes. The first theme was “Aspects considered by physicians in assessing prognosis.” When making a prognosis, physicians considered the following medical issues: nature and severity of disease, the role of treatment, course of the disease, external information, and medical evidence. Patient-related issues and physician-related aspects were also distinguished. Patient-related aspects concerned the patients’ work perspectives and coping or recovery behavior. Physician-related aspects concerned awareness of the physician’s own role and reflection on aspects such as empathy for clients and ethical considerations. The second theme was “Difficulties physicians face in assessing prognosis,” which included challenges during the assessment of diseases of a complex or less concrete nature, applying prognostic evidence to the individual, and lack of time when seeking prognostic evidence. The third theme concerned “Needs and solutions” formulated by physicians that facilitated the prognostic assessment. It consisted of continuous education, better collaboration with medical specialists and/or labor experts, and the use of prognostic tools such as checklists, apps or internet applications incorporating evidence on prognosis.

**Conclusions:**

Physicians identified several medical and patient-related aspects that elucidated the prognosis assessment. Given the variety of challenges and the need for further support found in the current study, future research should focus on the development and evaluation of training, tools, and guidelines to improve prognosis assessment by physicians.

## Introduction

Throughout the world, assessment of prognosis is an essential task of medical professionals working in the field of disability evaluation [1]. According to Moons et al. [2] “prognosis” means foreseeing, predicting or estimating the probability of risk of future conditions. Prognostic evidence helps in guiding clinical decision-making, improves understanding of the disease process, may define groups at risk based on prognosis, and predicts disease outcome [3].

The assessment of prognosis is relevant in curative medicine as well as in work disability evaluation [4]-[5]. In curative medicine, prognosis is related to the probability of an individual developing a particular state of health over a specific time, based on their clinical and non-clinical profile. Prognostic outcome helps the physician to make decisions about whether to intensify, change, or stop certain treatments [6]. At the same time, it allows the patient to prepare for any imminent consequences [6]-[7]. In work disability evaluation, making a prognosis assessment primarily entails the judgment of an individual’s functioning or capacity to work [8], which incorporates an assessment of the prognosis of their state of health. Prognostic outcome in disability evaluation is therefore related to improvement, stabilization or deterioration in functioning. Moreover, the provision and duration of the disability pension and, in the Netherlands, the level of benefits granted to patients, are dependent upon prognostic information and legislative rules, which also highlight its socio-economic significance [9]. Around the world, a variety of professionals, including insurance physicians, occupational physicians, general practitioners, disability evaluation physicians, and medical advisors are involved in this assessment. Although the setting, the insurance and legislative systems, and the clinical backgrounds of these professionals may vary between countries, work disability evaluations commonly use medical and non-medical information, such as demographic, personality, disorder-related, and work-related factors [10]-[11].

In work disability evaluation, the main perceived uncertainties and questions asked by physicians are of a prognostic nature [1]. Studies show that physicians in other specialties feel poorly prepared for assessing prognosis and find it stressful and difficult to make predictions [7],[12]. Physicians’ estimates of survival or risk of disability regarding various diseases are often inaccurate and systematically overly optimistic [13–15], meaning that decisional conflict is not uncommon in assessment of prognosis [16]. The inability to exclude perceived uncertainties creates challenges for physicians [17]. Little is known about the complex process of decision-making within the assessment of prognosis by physicians performing disability evaluations [1],[18]. Therefore, to make this assessment more explicit, the following research question was formulated: Which aspects are considered, and what difficulties, needs and potential solutions can be identified as affecting prognosis assessment by physicians performing work disability evaluations?

## Methods

### Design

We conducted a qualitative study using in-depth interviews to explore the nature and variety of aspects considered, as well as the difficulties and the needs and potential solutions that physicians identify as arising during the assessment of prognosis. In-depth interviews enabled us to gain more insight into their thought processes when assessing possible improvement or deterioration in functioning. Interviews allowed us to focus on a wide range of different aspects and personal experiences [19]. We reported our qualitative study based on the “Consolidated criteria for reporting qualitative research” [20]. The research was conducted in accordance with the Helsinki declaration [21]. The research proposal was submitted to, and approved by, the Medical Ethical Committee of the Academic Medical Center, which judged that a comprehensive evaluation was not required since this study was not subject to the Medical Research Involving Human Subjects Act (MEC number: W15_253 # 15.298).

### Recruitment and selection

We selected physicians working at the Dutch Social Security Institute: The National Institute for Employee Benefit Schemes (UWV)–who perform the majority of work disability evaluations in the Netherlands, including the assessment of prognosis. These physicians are medical specialists trained to evaluate work ability or disability of clients applying for a disability benefit.

We used a purposeful sampling approach to obtain an in-depth picture of the assessment of prognosis in daily practice by approaching a variety of physicians by email or phone, considering geographical location, gender, years of work experience, and the legal context of their main task. Before beginning, we estimated that based on these characteristics, between 20–30 physicians were needed to achieve data saturation.

### Interviews

The UWV was approached in three of the eleven regions of the Netherlands by contacting local management and medical staff. After permission was obtained, physicians were scheduled for a face-to-face interview of 60–90 minutes at the different agencies. In advance, they received an information brochure about the study. On asking, we provided them with additional information. After completing a written informed consent form, interviews were planned. Before we started, physicians completed a questionnaire on personal characteristics. The confidentiality of the data obtained and the privacy of the physicians were emphasized and guaranteed by using codes and removing all the demographic features referring to the interviewee.

Two researchers (RK and JH) attended and carried out all the interviews together. Interviews were audio recorded after permission was given. Both interviewers combined their experience in conducting qualitative research (JH) and in interviewing professionals and patients in clinical practice (RK) and in research (JH). The interviews were carried out from January to May 2015. To explore the process of decision-making within the assessment of prognosis the physicians were asked to consider and elaborate four questions:

- Which aspects do you consider in prognosis assessment?
- What difficulties do you have in prognosis assessment?
- What needs do you have in prognosis assessment in terms of skills, support or tools?
- What possible solutions do you have to address those difficulties during prognosis assessment?

Depending on the answers during the interview, the interviewers further explored responses to these questions by asking for examples that could elaborate statements made by the physicians. Our analysis started after performing half of the expected number of interviews. All physicians received a written transcript of the audio recording of their interview and were asked to check whether this was accurate were invited to add any other comments to the interview (member check). Saturation was determined complete when no new information was being drawn from the sample and this was confirmed by the research team.

### Analysis

A research assistant (MS) transcribed the recorded interviews and field notes verbatim. The data was managed using MAXQDA software (Verbi GmbH, Marburg, Germany). Using open coding, axial, and selective coding [22] we grouped codes with similar characteristics together into (sub)categories within overarching themes that corresponded with our research questions (aspects, difficulties, needs and potential solutions).

Throughout this process, the research team regularly met (RK, JH, JV, MS, CH, HW and MF) to discuss findings and, where necessary, redefine codes and categories based on consensus and to evaluate if data saturation was reached.

## Results

All physicians who were approached participated in the study and no physicians dropped out or refused consent. We continued data collection until saturation was achieved after twenty interviews. None of the physicians changed or added information after emailing the interview (member check). In total 20 physicians were interviewed who had a mean age of 50 years (35–62 years) and consisted of 13 males and 7 females, who had worked from between 1 and 30 years as a physician (mean: 15 years

Results are presented for the three primary themes describing the aspects, difficulties, needs and possible solutions (1–3). We distinguished several categories and sub-categories within each theme (see Table 1).

**Table 1:** Themes and (sub)categories.

**Theme 1. Aspects considered by physicians in assessing prognosis**
A. Disease or disorder
I. Nature and severity of disease
B. Treatment
I. Type of treatment
II. Treatment effect
III. Quality of treatment
IV. Alternative treatment options
C. Course
I. Insight into the course of the disease
II. Cause and disease maintaining factors
D. External evidence and information specialist/practitioner
I. Evidence from literature/guidelines/protocols
II. Information specialist/professional
E. Patient-related considerations
I. Clients own vision concerning recovery
II. Work perspectives
III. Indirect advantage of being ill
IV. Significance of work
V. Recovery behavior
VI. Coping regarding disease or changed role
VII. Social problems
F. Physician-related considerations
I. Perceived role
II. Empathy for the client/medical ethics
III. Influence of stakeholders
IV. Client observation and related physician impression
V. Anticipation of outcome
**Theme 2. Difficulties faced by physicians in assessing prognosis**
A. Nature of the disease, including:
I. Co-morbidity
II. MUPs and psychiatric disorders
B. Lack of time in assessment of prognosis
C. Value of evidence/information specialist
I. Translation of prognosis of disease into functional capacity
II. Inadequate answers practitioner
III. Incomplete diagnostics
IV. Validity of questionnaires in a claim situation
V. Experience with evidence-based medicine
VI. Translation of evidence into individual consequences for client
D. Consequences of informing the client about prognosis
E. Interpretation of legislative rules
**Theme 3. Needs and solutions of physicians in assessing prognosis**
A. “Instrument,” including checklists, helpdesk and databank B. Education
I. Renewing knowledge through internships in hospitals
II. Focus on treatment effects
III. Practice assessment of prognosis
C. Collaboration with labor expert, nurse practitioner, information exchange with other external disciplines

### Theme 1. Aspects considered by physicians in assessing prognosis

#### A. Nature and severity of disease

Physicians reported that the nature of a disorder was the starting point for assessing prognosis and that medical improvement was linked to improvement of functioning. Usually, assessment of prognosis was found to be very difficult. However, depending upon the nature of the disease, there were a few cases mentioned in which fewer difficulties were seen. For example, patients who had experienced a cerebrovascular accident would, in most cases, not experience dramatic improvement in their functioning after a two-year period. Others mentioned severe malignancies with poor survival rates, health conditions such as arthrosis and degenerative defects, clear-cut injuries without co-morbidity (e.g. fracture of the hip), or a disease with clear objective functional parameters, such as chronic obstructive lung disease, as examples in which assessment of prognosis was perceived to be straightforward.

In addition, psychosocial aspects were mentioned as complex disease maintenance factors influencing prognosis in a negative way, but also as aspects susceptible to improvement given the opportunities to intervene.

*An intervention from a psychologist to gain more regularity and structure*, *preferably making her active and socialize her again*. *[7; 144]*

*Someone is more than just their disease* … *you might also say that if the coping style is better than expected*, *you will also expect more recovery in terms of resilience [11; 32–34]*

Physicians also assumed a priori a favorable prognosis in patients with, for example, treatable stress symptoms or an uncomplicated disorder such as anemia.

Depending upon the severity of disease, physicians contemplated any current or potential intervention when assessing the chance of possible improvement. In some cases, they concluded that the patient’s health status would not improve, resulting in total and permanent inability to work, based on the stage of a disease and lack of therapy options:

*It was a colonic carcinoma metastasized to the liver*. *Had several operations*, *so that’s serious enough to easily rule out work completely (be declared completely unfit for work) [16; 7]*

#### B. Treatment

The physicians reported that evaluating the role of treatment is important during the assessment of prognosis, and they gather information from both the patients and practitioners about the type of treatment that has been given. Some also mentioned that this included thinking about the possible effects and aim (supportive or therapeutic) of these treatments.

… what are effective treatments, what are their results, ……does that actually really make a difference? …….you can also make your prognosis based on that [3; 262]

An appraisal of the effectiveness of treatment in relation to the literature or existing guidelines was included in their considerations, as was their familiarity and experience with the quality of the treating practitioner. Some reported considering possible alternative treatment options if current treatment or treatment in the past was not deemed to have been effective. A recurring but difficult question was whether someone had been optimally treated and, therefore, whether maximum improvement of functioning had been achieved:

*Yes*, *I think one of the most important questions is*: *has someone received optimum treatment*? *But in that case you also need to have an overview of all the guidelines for the standard treatments for each pathology*. *But I do think it is an important question* … *If it appears that there are no further options for treatment* … *that improvement (in terms of functional possibilities) can no longer be expected [6; 202–212]*.

Unless treatment was palliative, the physicians stated that a positive effect on prognosis was still possible as long as treatment was on-going. They reasoned that otherwise it would be senseless to continue treatment.

In the case of recovery from an underlying disease, the physicians also indicated that even if there were remaining functional impairments, it might still be possible to achieve improvement of functioning; for example, through rehabilitation:

*… you have a fracture*, *healed after three months but* … *…*.*limitations still continue…*. *how will he learn to deal with that*? …. *it depends on rehabilitation… it is still possible to function effectively even with serious limitations [5; 83]*.

#### C. Course

Much value was attributed to information obtained during previous consultations with physicians, giving more insight into the course of complaints, the possible effects of various treatments over time, improvement or worsening of functioning, and presentation of the patient. The physicians distinguished between recovery, stability or progression of disease by focusing on the course of the disease, as well as the functional, personal, and environmental aspects. These factors relating to the course of the disease might occur spontaneously or be influenced by therapy, as was stated by one of the physicians. Especially if there is a lack of recovery, some physicians investigate the causes of this, aiming to advise a tailored treatment or intervention, and influence the course of the disease in a positive way. Assessment of the course of vocational rehabilitation was another feature mentioned as important in relation to prognosis. The observation that a patient with some disability is not able to further expand their activities and that the level of functioning has consolidated, provides the physician with information about prognostic outcome:

*…At that moment*, *he was back in some form of reintegration*. *He was working …and was doing his work satisfactorily*, *which for us was another reason to say*: *this is a stable condition…that is permanent [5; 55]*.

#### D. External evidence and information from treating specialist/practitioner

Physicians often rely on their experience as a disability expert in assessing prognosis. However, in cases where a solid, substantiated argument is needed, or when they are in doubt, they reported that they would use prognostic information from guidelines or protocols. Google (Scholar), PubMed, and medical journals were mentioned as sources of information for the assessment of prognosis.

*…*.*Well*, *if you have good evidence …then I might have said … that the literature suggests it is a condition that can be extremely disabling*, *but for which there is more than 50% chance of recovery within 1 to 2 years* … *In that case*, *it cannot be called permanent disability* [20; 248–251)

Another source of information regarding diagnosis, treatment, and prognosis came from treating medical specialists or general practitioners. Some physicians valued medical information from treating medical specialists as their main source of information. Frequently, this information informed physicians about whether improvement was to be expected given the potential treatment options still available to the patient, or whether the opposite was more likely and all treatment options had been exhausted.

#### E. Patient-related considerations as perceived by the physicians

Physicians regarded the opinion of patients concerning their recovery, or the patient’s perspective on their future and motivation with respect to returning to work, as an important predictive factor that can positively or negatively influence prognosis. In some patients who were not motivated to return to work, or were expecting to lose income because of difficulties experienced in returning to work, physicians observed a focus on limitations and an inclination not to feel better. The physicians considered this mechanism–the indirect advantage of being ill–a negative prognostic factor.

*People’s own expectations…they are an important factor in how much effort there will be towards reintegration and seeking work……what are the implications of losing benefits for this client*?.....*in that case*, *part of the prognosis…focusing on the limitations……*.*will ultimately determine the client’s behavioral response to their sickness [5; 171–197]*

The physicians also considered work itself as a positive prognostic factor enabling the patient to gain confidence and subsequently improve their own functioning in a positive way by developing self-esteem.

*Currently*, *her position is that she will need reasonably intensive support* … *even at work* ....*And so*, *as she becomes more secure and gains more self-confidence* … *the need for support can gradually become less intensive*.

An adequate coping style and the role of recovery-enhancing or other behavior were mentioned as explanations of why some patients had improved or not. Finally, stressful or troublesome psychosocial aspects were mentioned as associated with a poor prognosis, such as poor living circumstances of patients, domestic problems, financial debts, and the absence of social support.

#### F. Physician-related considerations

Physician-related characteristics were mentioned as aspects that could potentially influence the prognosis assessment and prognostic outcome, although it was not clear to what degree, or in which direction. The physicians reflected on their own role in prognostic assessment, particularly in cases where the decision was difficult or when their decision had important consequences for the patient. Some physicians explained that they applied prognostic information strictly from guidelines or strictly implemented the legislative rules that impacted on prognostic evaluation. However, they sometimes also allowed themselves freedom of interpretation in accordance with what they deemed fair. A frequently mentioned example was the hesitancy with which they would declare a young person permanently unfit for work, as, from a social point of view, this would exclude them from employment and society.

As a result of patient-doctor interaction, feeling empathy or lack thereof was indicated as another aspect for consideration. If a physician had experience of a similar disease to the patient, this resulted in better understanding of the patient’s complaints and opinion. In these cases, the physicians were more inclined to agree with the patient’s opinion, thereby influencing prognostic outcome. In some cases, feeling empathy meant that although the prognosis was considered poor (e.g., in patients with terminal cancer), the small chance of recovery was emphasized in order to give the patient hope (that they are doing well) and positively influence their aspirations of returning to work. The physicians also reflected on medical ethics, especially in relation to not wanting to harm the patient:

*…*.*So what about dealing with ethical factors as a doctor…… a physician should not cause harm*, *but do good*, *…*.. *what are the arguments for and against …*..*then*, *you have clearly identified your own internal process of deliberation and how you came to your decision (prognosis)…*. *[10; 275]*

The ability of physicians to resist the wishes of employers or the prevalent opinions of their fellow physicians were other aspects taken into consideration, again in relation to influencing prognostic outcome and sometimes in opposition to intuitions.

*…An employer who gives a clear impression …*..*that they want rid of someone as quickly as possible*. *…*.*In that case*, *the interests of an individual*, *the employee I have to assess*, *are set against the interests of a business*. *In that case*, *a lot of it depends on your personal standards and values as to whether you go for an individual or for a business*. *The consequence of the prognostic outcome then becomes part of the decision-making process [1; 101–103]*.

In addition, the patient’s presentation during consultation was reported as being an important aspect in enabling the physician to weigh the severity of the problem or link this information to plausibility and consistency of the disease, and subsequently translate these findings into an appropriate prognosis.

Finally, the physicians reflected on anticipating the outcome for patients. For example, this might occur in the case of declaring that someone will not recover further, with the physician considering the consequences that this might have for the patient regarding participation in the labor market.

### Theme 2. Difficulties faced by physicians in assessing prognosis

#### A. Nature of disease

Not knowing the cause of a complaint, feelings of uncertainty, or dealing with a less concrete syndrome or condition, as is the case in co-morbidity, medically unexplained physical symptoms, or severe psychiatric disorders, were mentioned as barriers to prognostic evaluation:

*Pathologies where you do not have a clear idea of what the cause is* … *How does it develop*, *how is it to be treated*? *…*.*In the case of pain syndromes*, *for example*. *And then I have to give a response on the issue of how permanent it is*. *In that case I say*: *at the moment*, *I do not think much more can be expected*. *But then I will always still have some doubt [3; 169]*.

#### B. Lack of time

The physicians reported that one of their tasks was to examine their findings carefully and elaborate on their conclusions in disability evaluation. They must complete comprehensive reports, and lack of time was regarded an important barrier to further consultation of guidelines, searching PubMed, consulting other practitioners, and subsequently producing a substantiated prognostic assessment:

*I tend not to delve into all of the literature because it takes so much time [8*; *185]*

*…*.*Searching Pubmed ………really does take an awful lot of time [15; 180–182]*.

#### C. Weighing of information from a medical specialist or value of evidence

Although physicians mentioned evidence or medical and prognostic information from a medical specialist as the leading source of information used in the assessment of prognosis, they also recognized various difficulties with respect to evaluating and weighing up such information. Prognostic information from practitioners is often related to disease and not to functioning. Sometimes the information received was too general or had no relationship with the information requested by the physician. Occasionally, medical specialists did not respond to requests for medical information about claimants. Sometimes diagnostic information was incomplete, making it difficult to assess the prognosis in relation to the severity of a complaint:

*So*, *this gentleman suddenly has blood in his urine* … *making a prognosis in that case is of course extremely difficult if there are all kinds of symptoms that have not yet even been tested for and you do not yet have any idea what the diagnosis is*. *If he’s peeing blood*, *he could have kidney cancer*, *I don’t know…*.. *Have there been diagnostic tests*? *If the hematuria is not caused by kidney cancer*, *was it non-recurrent and of unknown cause*, *that is extremely advantageous in terms of the prognosis [8; 124–136]*.

Translation of evidence from guidelines or the medical literature to the individual patient was perceived as difficult and a significant obstacle:

*The ongoing difficulty … is that you are always making an individual assessment in the case of a particular client in front of you*. *And that makes it even more difficult to apply the protocol which leads to a general pronouncement [10; 77–77]*

Some physicians had doubts about the reliability of questionnaires, particularly in cases of mental health disorders, questioning whether, for example, symptom questionnaires that provide insight into the severity and/or presence of various disorders are validated tools for use in assessing claimants. A lack of experience in searching for evidence is another hurdle in the assessment of prognosis. This may lead to the neglect of available evidence.

#### D. Consequences of informing the client about prognosis

According to the physicians, communicating to the claimant that there is only a small chance of improvement in functioning would not stimulate an active attitude toward recovery in a patient, thereby affecting the already poor prognosis in an even more negative way. The obligation to inform the patient of the possible negative outcome is experienced as an ethical dilemma. In contrast, when the prospects looked good from the perspective of the physician, they believed that giving the patient hope would affect recovery behavior in an even more positive way.

*… a prognosis …*.*that it will be permanent and never get better makes it very likely that you are not encouraging him to do something about his health…*..*but I still tell him…*. *Because I want to be clear about things to the client and also because I want to make clarify my own considerations and assessments*. *Simply being open in contacts with the client while also pointing out that there may occasionally still be hope beyond the horizon*. *[10; 51–57]*.

#### E. Interpretation of legislative rules

The way in which legislative rules are formulated was reported as an obstacle in the assessment of prognosis. Within the Dutch social security context, physicians working for the UWV have to make a clear distinction concerning whether a patient’s functioning is stable and improvement is impossible, or whether any improvement is likely in the future. The physicians indicated difficulties in applying such legislative criteria, not only questioning the definition of improvement, but also the translation, in terms of risk percentages:

*Improvement in functioning …*.. *None or hardly any…*.*by the way*, *what percentage is that again*?..... *So I think it is definitely less than 5%*. *Colleagues have different opinions*, *we sounded them out again*. *The boundary is somewhere around 20%*, *there are some people who would say that 20% is definitely more than none or hardly any [12; 85]*

### Theme 3. Needs and solutions of physicians in assessing prognosis

The physicians reported several aspects that could support them in assessing a patient’s prognosis, without making a clear distinction between needs and solutions. Upon asking whether they could classify these, they distinguished three categories: “tools,” “education,” and “collaboration.”

The physicians suggested that a list of drug-related treatment effects or an outline, list, or database presenting various diseases and the related recovery time after adequate treatment would be supportive and convenient to use in the assessment of prognosis.

*That would definitely be useful* … *Being able to have some kind of database for a number of common disorders*. *We can quickly check what the effective treatments are*, *what the results are and in what percentage (of cases) it actually makes a difference*. *[3; 254–262]*

In addition, they also suggested a list of positive or negative prognostic predictors or, in the case of malignancies, survival rates would be useful.

*If you have a list……there are prognostic factors that prevent further recovery or have a negative predictive value…*.*such as low level of education*, *psychosocial stress factors*, *you name it*, *working relationship*, *sickness*, *spouse*, *children*, *and so on* … *[16; 227–229]*

Furthermore, they stated that information about the possibilities of changing disease-related functional limitations by means of an intervention (e.g., graded activity) could facilitate the prognostic assessment in a positive way. In the case of a patient’s reassessment, access to comprehensible prognostic information from earlier reports and clarifying considerations that provide insight into former judgments was believed to be helpful. Moreover, guidance provided by a helpdesk, where an expert can be consulted and provide detailed answers on prognosis and evidence on specific prognostic questions–for example, concerning rare diseases–was also considered useful.

A database of prognostic evidence that can be readily consulted was also mentioned, such as a webpage containing links to guidelines or protocols regarding prognostic issues. In a more sophisticated format physicians proposed an interactive internet application including a database option to allow specific patient and disease characteristics to be entered. Physicians mentioned that such an application ideally provides specific individualized prognostic information (evidence, risks) and ensures that all prognostic decisional aspects are considered in consecutive steps.

*a database that includes everyone*. *And everyone is listed*, *with name*, *gender*, *date of birth*, *that is just basic*. *Alongside that*, *you had …*..*a problem list*. *So anyone known to have hypertension*, *diabetes*, *was included in the problem list…* … *And alongside that …*..*an entire system with folders for when people have tennis elbow* … *what someone should do themselves to recover from that*. *…* … *…*..*So then you could give a recommendation……standards were included…(with average clinical course and prognostic factors)*..*……you could click on them based on that diagnosis code* … *that would certainly provide me with support [20; 264–282]*

In general, physicians emphasized that any support tool must be inviting to use, practical, quick to consult, and easily accessible.

In relation to bringing prognostic knowledge to the required standards, much value was attributed to education and refresher courses on prognosis. Post-graduate courses, evidence-based medical training, renewing knowledge and experience through internships in hospitals, or simply asking for feedback from colleagues or practicing the assessment of prognosis using case histories, were given as examples.

*Lots of different things could make a contribution……discussing reports in small groups with colleagues …*..*it would also help if we could have refresher courses based on case histories…*.*do e-learning…*.*provide lessons about it…*.*practice*, *practice*, *practice…*.*telling people more about the prognosis…*..*backed up by evidence [18; 161–191]*

Collaboration with another professional working in the area of disability evaluation was emphasized, for example, with a labor expert. Within the disability evaluation context in the Netherlands, a labor expert considers whether the patient’s functional limitations, as assessed by the physician, are compatible with work adjustments. As some, but not all, functional limitations may improve over time, a labor expert can indicate whether this improvement will result in future work opportunities that are currently not possible. If that is the case, this information may allow physicians to not judge a patient permanently unfit for work.

Exchanging medical information and debating prognostic issues with colleagues in other medical disciplines, thereby gaining more insight, was also reported as a facilitating aspect. Finally, support personnel such as nurse practitioners were seen as helpful, contributing to time efficiency and complementing the physician’s work, especially with respect to the collection of prognostic information prior to the disability evaluation. According to one of the physicians, having this information, especially regarding non-medical aspects, helped complete the patient’s prognostic picture and therefore facilitated prognosis assessment.

## Discussion

This study showed that prognostic assessment is perceived as a complex process by physicians and not only includes assessment of medical aspects such as the nature and course of a disease and the role of treatment, but also the role of patient-related aspects such as the patient’s view on return to work, or aspects such as coping or recovery behavior. The physicians often reflected on ethical dilemmas and their own role and perceptions, such as having empathy for clients. Difficulties were encountered during the prognostic assessment of psychiatric disorders, medically unexplained physical symptoms, or cases of co-morbidity. More practical challenges included lack of time, difficulties in interpretation of legislative rules, and challenges in translating evidence to the individual patient’s condition. In daily practice, the physicians often assessed prognosis based on their own opinions, experience or training. Our study also provides insight into how physicians would like to be supported during their prognostic assessment, that is, with the assistance of online tools, continuing education, and information exchange with other medical specialists.

Performing individual interviews helped to provide in-depth information on the prognostic assessment process. In this study, the two interviewers complemented each other, which fostered in-depth questioning and greater reflection. The participants’ differences in demographic and other characteristics, such as geographical location, gender and experience, resulted in a broad spectrum of physician opinions. However, given the specific legislative and social security context in the Netherlands, which may have an impact on long-term outcomes such as return to work, the current findings may not be generalizable to an international level.

In addition, the use of prognostic evidence, such as professional guidelines, was only mentioned by a few participants. In the present study, our selection resulted in the inclusion of participants with, on average, 15 years’ experience (range 1–30 years) and only a few had recent training in evidence-based medicine (EBM). Medical training (including post-graduate) in the Netherlands has an increasing focus on teaching academic skills, such as searching for and applying evidence. Thus, we expect the role of prognostic evidence to increase over time as knowledge and skills in EBM increase, and an adequate knowledge infrastructure for physicians becomes available [4].

### Comparison with other studies

#### Prognostic process, aspects and prognostic outcome

Specific evidence on the process of prognosis assessment during disability evaluation is limited. The unpacking of this prognostic process could make a valuable contribution to a structured prognostic judgment, possibly reducing differences in prognostic outcome. The physicians in our study stated that the aspect of “Nature of a disease or disorder” is generally the starting point in the evaluative process both for short and long-term term prognosis assessment [23]. In other studies on disability assessments by physicians, similar aspects that were considered important in prognosis assessment included socio-medical history, treatment aspects, and/or vocational interventions [24]-[25]. However, physicians’ understanding of these aspects and the value they attach to them may differ, and thus subsequently affect prognostic decisions/outcomes. Agreement between physicians on prognostic outcomes is thus challenging [25]-[27].

A study in several European countries showed that physicians were unable to achieve agreement on the provision of a disability pension, even when adequate interventions had been provided [26]. Similarly, in national guidelines for physicians in various European countries and the US, differences in expectations about the duration of sickness absence were identified for similar diseases [27]. Differences in legislative rules may impact the prognostic assessment process across countries [24], influencing prognostic outcomes. Our study complements the important research [28] that has shown that physicians’ norms and values influence prognosis and may contribute to inter-physician variation in prognosis assessment. As there is a subjective dimension in the way assessors collect and interpret information [29], prognostic decisions/outcomes may differ. Differences in prognostic and disability judgments may be a consequence of variations in interpretation, understanding, values, and use of different criteria [29]-[30]. Research shows that better agreement can be reached using training and instruments to standardize the collection, interpretation, and reporting of information [30]. The systematic evaluation of various aspects of the prognostic assessment which was undertaken in this study aimed to further develop prognostic aids and training solutions. This could be the first step in developing an improved, more evidence-based assessment of prognostic outcomes in work disability evaluation.

#### Medical aspects and patient-related aspects

We found that the prognostic aspects mentioned by our participants to a large degree resembled the aspects used by physicians working in curative medicine, such as type and severity of illness, functional status (activities in daily living), patient symptoms, and psychosocial elements [31]. The focus on medical aspects during the assessment of prognosis, also in disability evaluation, may be due to the basic medical training that doctors receive in medical school [12], [32]. In addition to medical aspects, inadequate coping, a lack of self-efficacy, and negative perceptions are also assessed by physicians in various countries when undertaking work or general disability assessments [33–35]. The presence or absence of these patient-related aspects may promote or hinder return to work [11], [33], [35]. The physicians in our study also attributed value to patient-related factors that can influence a person’s motivation or ability to work in the future. A focus on the patient’s work perspectives, coping or recovery behavior was considered especially important, as these factors can be targeted and potentially improved by interventions such as counseling or other psychological therapy. Although it is unclear to what extent interventions can influence these patient-related aspects of work disability evaluation with the aim of improving a prognostic outcome (RTW), targeting and intervening in patient-related or psychosocial aspects, in addition to the medical aspects, may be beneficial.

#### Ethical aspects

Like other studies in clinical practice [36], physicians in our study frequently struggled when discussing the prognosis with their patients, being concerned about the emotional impact of communicating a negative prognosis. Concerned with causing emotional distress and thereby influencing the prognosis in a negative way, they sought a balance between making definitive prognostic judgments and more indeterminate statements. The et al. [37] showed that both physicians and their patients tend to communicate about short-term treatment activities and check-ups but neglect long-term outcomes. It has been found that neglecting a discussion of the inevitable long-term outcome keeps patients hopeful and optimistic [37]. This finding may be related to ethical principles that guide the professional behavior of physicians, such as doing good (beneficence) and not harming patients (non-maleficence) [38]. As work participation has been linked to improvements in quality of life and life expectancy [39], this may explain why the physicians in our study communicate the positive prognostic consequences of work, attempting to motivate patients to participate. Similarly, they were reluctant to declare young people permanently unfit for work despite a poor prognosis, as this denied them the potential positive effects of participating in future work. Promoting work participation or being reluctant to conclude someone is ‘permanently’ unfit for work might also reflect the physicians’ desire to do good and prevent harm.

Prognostic uncertainty, difficulties and potential solutions in prognosis assessment Given the complexity of prognostic assessment, a certain amount of uncertainty in the physician is inevitable. However, complexity and uncertainty are not limited to the field of disability evaluation, having also been reported by experienced healthcare physicians [17], [40]. Uncertainty has been reported as a result of knowledge deficits or from the variety in patient and disease presentations [41]. In our study, uncertainty was reflected in the physicians’ hesitation about whether they should strictly apply legislative rules or diverge from them and handle the case more “in the spirit of the rules.” In addition, indefinable aspects, such as experience or intuition, may create uncertainty; for example, when the course of a disease does not follow a familiar pattern [40]. Certainly, the course and patterns of disease seen by physicians who perform work disability evaluations are complex, and co-morbidity is often present.

Furthermore, physicians perform disability assessments in a context in which conflicts of interest are present [42]. The outcome of prognosis assessment may influence the financial benefits granted to claimants. Thus, on the one hand, they are asked act as gatekeepers to the social security system, while on the other they have to consider patients’ interests. Professional guidance in the form of guidelines is frequently absent, difficult to interpret or translate in relation to individual cases, and requires that physicians use their experience. Pontin and Jordan [40] suggested several solutions to deal with uncertainty, such as the possibility of a second opinion or assessing patients several times over a longer period. These solutions may also be feasible in the field of disability assessment.

As our physicians clearly expressed the need for adequate training in prognosis assessment, we also see opportunities for improvement here. Rogg et al. proposed that physicians should improve their skills and knowledge in assessing prognosis by using tools, training, and refresher courses [43]. Only a few physicians reported that they used guidelines, protocols or lists of prognostic factors in their prognostic assessment. Supporting physicians in applying prognostic evidence in individual cases is important, as it encourages the use of evidence in the field of disability evaluation [4] and curative medicine [44].

More training in evidence-based practice for physicians has been shown to enhance the use of evidence, increase knowledge and skills in evidence-based medicine, and improve self-efficacy in the field of disability evaluation, which includes the assessment of prognosis [45]. The prognostic uncertainty felt by our participants may lead to greater receptiveness to the use of evidence-based prognostic tools in the future [46]. The development of prognostic evidence and prognostic tools has been widely recommended in medicine [47]. As indicated by our physicians, such a tool must be inviting to use, be practical, quick to consult, and easily accessible. Our respondents also attributed much value to frequent reassessments, which can monitor the course of both disease and functioning. Guidelines recommend regular consultations or reassessments in, for example, mental disorders or low back pain, showing significant positive effects on return to work [48]-[49]. In addition, facilitating collaboration between physicians performing work disability evaluations and other physicians or labor experts may aid a better exchange of prognostic information.

Finally, the present study identified several medical and patient-related factors which elucidate the assessment of prognosis by physicians involved in work disability evaluation. Given the variety of challenges and the need for further support expressed by the physicians who participated in our study, future research should focus on the development and evaluation of training, tools, and guidelines to improve prognosis assessment by physicians.
